# In-vivo coronary flow profiling based on biplane angiograms: influence of geometric simplifications on the three-dimensional reconstruction and wall shear stress calculation

**DOI:** 10.1186/1475-925X-5-39

**Published:** 2006-06-14

**Authors:** Ernst Wellnhofer, Leonid Goubergrits, Ulrich Kertzscher, Klaus Affeld

**Affiliations:** 1 German Heart Institute of Berlin, Berlin, Germany; 2 Biofluid Mechanics Laboratory, Charité-Universitätsmedizin Berlin, Berlin, Germany

## Abstract

**Background:**

Clinical studies suggest that local wall shear stress (WSS) patterns modulate the site and the progression of atherosclerotic lesions. Computational fluid dynamics (CFD) methods based on in-vivo three-dimensional vessel reconstructions have recently been shown to provide prognostically relevant WSS data. This approach is, however, complex and time-consuming. Methodological simplifications are desirable in porting this approach from bench to bedside. The impact of such simplifications on the accuracy of geometry and wall shear stress calculations has to be investigated.

**Methods:**

We investigated the influence of two methods of lumen reconstruction, assuming circular versus elliptical cross-sections and using different resolutions for the cross-section reconstructions along the vessel axis. Three right coronary arteries were used, of which one represented a normal coronary artery, one with "obstructive", and one with "dilated" coronary atherosclerosis. The vessel volume reconstruction was performed with three-dimensional (3D) data from a previously validated 3D angiographic reconstruction of vessel cross-sections and vessel axis.

**Results:**

The difference between the two vessel volumes calculated using the two evaluated methods is less than 1 %. The difference, of the calculated pressure loss, was between 2.5% and 8.5% for the evaluated methods. The distributions of the WSS histograms were nearly identical and strongly cross-correlated (0.91–0.95). The good agreement of the results was confirmed by a Chi-square test.

**Conclusion:**

A simplified approach to the reconstruction of coronary vessel lumina, using circular cross-sections and a reduced axial resolution of about 0.8 mm along the vessel axis, yields sufficiently accurate calculations of WSS.

## Introduction

Based on the hypothesis that information on local wall shear stress (WSS) patterns has a prognostic value with respect to the progression and risk of coronary artery disease, in vivo profiling of the endothelial shear stress in coronary arteries has been performed recently in several studies [1-7]. Published serial invasive investigations in the last years support the prognostic impact of local WSS evaluations [8,9]. Most of these studies were performed by three-dimensional (3D) reconstruction of coronary artery segments fusing intravascular ultrasound images (IVUS) and angiograms with subsequent numerical flow simulation studies. The IVUS is used because it provides detailed information of the circumferential endo-luminal border and also additional information of the vessel wall that is derived from imaging the local plaque. Numerical flow simulation is used since measurements of velocity profiles, and especially of wall shear stress distribution in the coronary arteries, are not feasible.

3D-reconstruction of coronary artery segments fusing IVUS-images and angiograms with subsequent numerical flow simulation studies is an invasive, expensive and time consuming approach that is limited to dedicated studies and rather small numbers of investigated vessel lesions. Further limitations of this method are size of the segments (> 1 mm), which may be assessed by the IVUS catheter. Thus the assessment of side branches and distal coronary arteries is not feasible. We do not know whether the costs of the accuracy of the reconstruction approach translate into the amount of additional clinical diagnostic impact. Simpler approaches may provide clinically relevant prognostic information [9]. Thus, research on simpler techniques is required. Furthermore, we would like to characterize coronary artery disease in the coronary trees as is shown in figures 2 and 3. We propose to use 3D-reconstruction of coronary trees based on biplane angiograms with subsequent numerical flow simulations.

**Figure 1:**
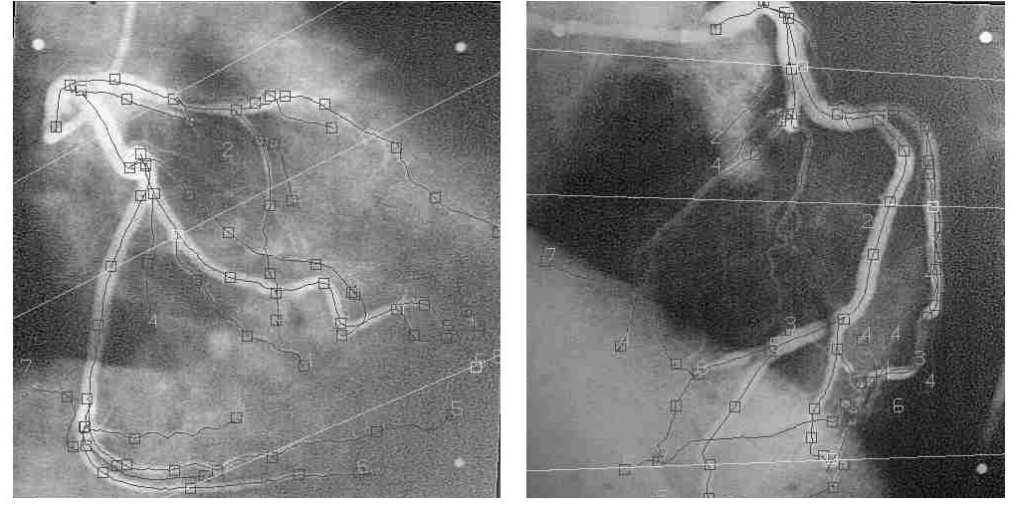
Example of a pair of corresponding LAO and RAO projections. The three straight lines in each image show corresponding projection lines in the two projections. These lines are used to match the two projections. Numerated squares mark nodes used for segmentation and identification of vascular branches.

**Figure 2:**
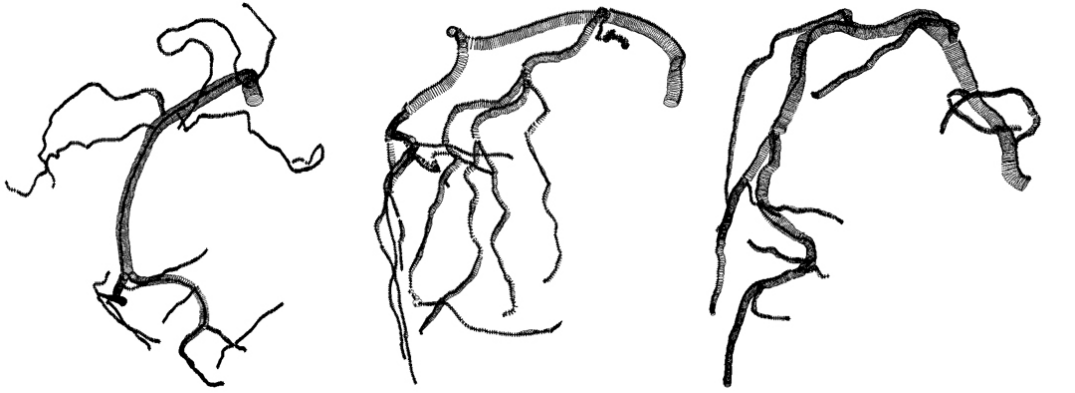
Wire representation of the three right coronary arteries investigated in our study. From left to right: normal right coronary artery, right coronary artery with "obstructive" atherosclerotic disease and right coronary artery with "dilated" atherosclerotic disease. Circular cross-sections are supposed. The reconstructions were done by use of the software Gambit™.

**Figure 3:**
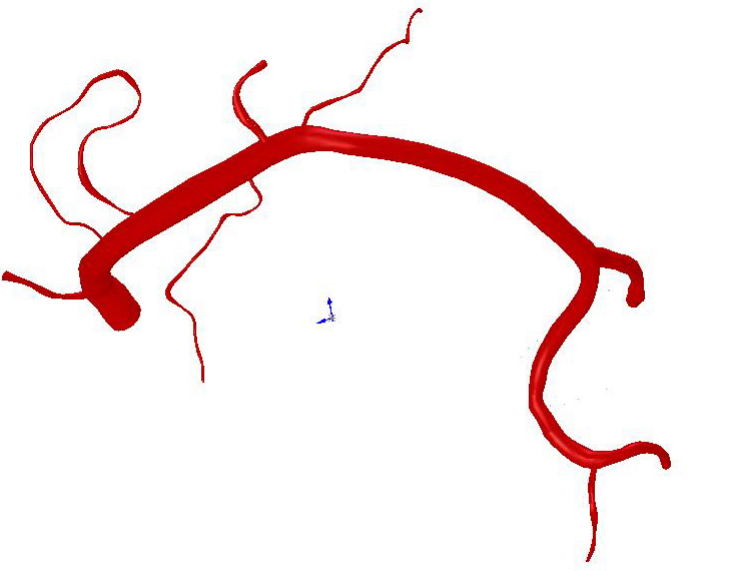
Geometry of the reconstructed endo-luminal surface of the normal coronary artery using Method 1 and the software SolidWorks™. A normal right coronary artery is presented.

Coronary artery disease is clinically diagnosed by symptoms related to impaired myocardial perfusion and invasively diagnosed by detection of wall irregularities or local obstructions in selective angiograms (luminograms). The luminal contour is the net result of the encroachment of plaque into the vascular lumen and compensatory vessel wall remodeling [10,11]. Luminal remodeling is localized and preserves a circular lumen even in the majority of eccentric atherosclerotic lesions [12]. The irregular lumina occur rarely, generally at severely diseased sites. These advanced lesions imply a failure of local remodeling. This in turn suggests a loss of endothelial function and of WSS responsiveness [13]. Luminal geometry and flow determine wall shear stress. IVUS-data shows that wall thickness and wall composition have no direct impact on WSS. Thus, wall shear stress estimation from standard luminograms using fast semi-automatic geometric reconstructions and flow calculations should be feasible and may be an important step from bench to bedside in providing clinically relevant WSS-data in vivo.

The proposed approach implies simplifications of geometric reconstructions and model assumptions with respect to flow simulation in geometries reconstructed from biplane angiograms. A methodological investigation of the impact of these simplifications on WSS calculations using in vivo data is necessary. The goal of this paper is an assessment of the impact of two different time saving simplifications of geometric reconstruction on cycle-averaged WSS. Furthermore, a new method of WSS distribution characterization is proposed – analysis of WSS histograms. A further goal of this paper is to investigate the impact of reconstruction simplifications on the proposed method of the WSS characterization.

## Methods

### Data

Three distinct cases of coronary artery luminal geometries reconstructed from biplane patient angiograms were used: a normal right coronary artery (control), and right coronary arteries (RCA) with atherosclerosis with "dilated" versus "obstructive" remodeling. The concept of "dilated" versus "obstructive" coronary atherosclerosis was introduced by Schoenhagen et al. [14,27]. The concept of Schoenhagen addresses the fact that remodeling is an important factor affecting the luminal width which is only loosely related to plaque growth. He says: "Traditionally, the development of coronary artery disease was described as a gradual growth of plaques within the intima of the vessel. The outer boundaries of the intima, the media and the external elastic membrane, were thought to be fixed in size. However, histologic studies demonstrated that certain plaques do not reduce luminal size because of expansion of the media and the external elastic membrane during atheroma development. This phenomenon of "arterial remodeling" was confirmed in necropsy specimens of human coronary arteries. [14]" Even though in his review Schoenhagen focuses on local remodeling assessed by IVUS and IVUS-specific definitions of remodeling, he sees a clinical analogy between positive remodelling and coronary ectasia. We apply his concept on atherosclerotic coronary ectasia ("dilated") and non-ectatic coronary artery disease ("obstructive"). The underlying hypothesis of our choice of these three particular coronary arteries is that vascular remodeling is intrinsically related to atherosclerotic inflammation and affects environments at multiple sites rather than localized foci. Thus profiles of WSS within whole segments or vessels might identify different patterns of remodeling associated with characteristic changes in the distribution of WSS. This is why three different vessels were used to study the impact of reconstruction simplifications on WSS characterization.

### Three-dimensional reconstruction

Biplane angiograms (25 frames/s) had been acquired on cine-film by a standard biplane angiographic X-ray device (Philips DCI-System) during end diastole. Rotation and angulations of the C-arms, distances of image intensifiers to X-ray sources and size of image intensifiers had been recorded. Frame numbers were used to find the corresponding images in the left anterior oblique (LAO) and in the right anterior oblique (RAO) projections. These protocol data were used to estimate the three-dimensional geometry. A two-dimensional (2D) model was reconstructed for each projection by a combination of interactive topology marking and automatic vessel detection. Vessel bifurcations were manually identified and used for both segmentation and vessel detection (see figure 1). 2D data is organized in segments consisting of coordinates of centerline and related radii, defining both edge points of the vessel projection for the corresponding 2D models of LAO and RAO projections. The 3D reconstruction is calculated from 2D projections in the three-dimensional space estimated from protocol data. Each data set representing a particular 3D reconstruction segment consists of a discrete set of vector triplets that represent the 3D coordinates of the segment centerline and the two radii R1 and R2 obtained from two projections. For a more detailed description of the 2-D models and the 3-D reconstruction procedures, please refer to [15, 16]. The accuracy of the used reconstruction software was tested and validated in phantom studies with well defined geometries and were described elsewhere [17]. The diameter and volume measurements in this model were performed by three-dimensional calipers (spheres and generalized cones). The resulting accuracy yields an error for diameters of <3%. The inter- and intra-observer variability is <5%. In these phantom studies a reconstruction procedure using elliptical cross-sections was applied.

There are two simplifications suggested by these considerations that speed up the reconstruction and circumvent the reconstruction problems with vector triplets representing degenerated ellipses and non-orthogonal (tilted) and unevenly spaced cross-sections. The first one is a reduction of the spatial resolution by using only every second vector triplet for interpolation. The cross-sections defined by the original vector triplets are tilted, because the radius vectors are not orthogonal to the longitudinal axis of the vessel. Thus neighboring cross-sections may intersect and unevenly spaced tilted cross-sections occur. The analysis of the 3000 cross-sections of the coronary artery represented in figure 2 left demonstrated that the mean angle between cross-section planes defined by vector triplets and longitudinal vessel axis was 90° ± 6.5° (SD). However, some cross-sections were angulated by less than 30°. A 3D caliper approach is necessary to construct cross-sections orthogonal to the longitudinal axis from the original vector triplets. As most of the cross-sections defined by vector triplets are nearly orthogonal, an approximate solution is to neglect one of two intersecting cross-sections or respective vector triplets. The axial resolution is only slightly reduced by this approach. The resulting models were defined as high fidelity models (HF). Two further resolution models were also studied – low fidelity models (LF) with a relative to HF models halved number of reconstructed cross-sections and double low fidelity model (DLF) with a relative to LF models halved number of cross-sections. The provided 3D reconstruction data defines only 4 points on the vessel cross-section. Interpolation algorithms are necessary to reconstruct surface and volume geometry and to construct a sufficiently fine grid of this vessel lumen for CFD study.

Although the biplane views were chosen orthogonal or near orthogonal, the radii are not orthogonal due to foreshortening. Vector triplets may even represent ellipses with high eccentricity. The data provides only an estimate of the real vascular eccentricity, however, since vessel foreshortening causes fake eccentricity. The analysis of the 3000 cross-sections reconstructed for the coronary artery presented in figure 2 left showed that the mean eccentricity of elliptical cross-sections, defined as the relationship between the two radii R1 and R2, was relatively small with 1.13 ± 0.34 (SD). The assumption of a circular lumen is supported by this data and data from IVUS studies that report the preservation of nearly circular lumina due to highly localized remodeling [12]. The second simplification is the assumption of a circular cross-section (Method 1) instead of an elliptic one (Method 2). These two methods proposed for cross-section reconstruction procedure are described in detail below:

#### Method 1 (circular lumen)

The radius R of the circles defining cross-sections was chosen as the geometric mean radius (R = (R1·R2)^1/2^). In this case, the cross-sectional area of the resulting circle is equal to the area of the ellipse where the major and minor axes equal to the two radii (R1 and R2). To convert data with original radii into the circular model the radii vectors R1 and R2 had to be scaled by factors R/R1 and R/R2 respectively. This calculation was done automatically using a macro written in MS Excel™. With the macro, obtained data is rewritten in a journal file format. This allows an automatic generation of all cross-section circles in 3-D-space with three defined points (center point and two points on the circle) by the software Gambit™ (Fluent Inc., Lebanon, USA). Figure 2 shows the resulting wire grid reconstructions of the coronary lumina. The resulting geometries were subsequently exported in commonly used IGES format.

#### Method 2 (elliptic lumen)

These vectors can be used directly as the axes of elliptic contours only in the case of orthogonality of two radius vectors. Otherwise, the shape of the ellipses is ambiguous due to a loss of spatial information. Ambiguity increases with a decreasing angle between the two radii. Three specific points are needed for an unambiguous definition of elliptic contours in 3-D space. The first point is the center of the ellipse. The second point together with first point must define the major axis of the ellipse, whereas the third point is located on the elliptical contour and does not coincide with the second point. Since both radius vectors were defined using the projection edges of vessel lumina, each may initially be considered as the major axis. If neither of both projections is orthogonal to the major axis of the real elliptic cross-section, then neither of the radius vectors represents the direction or length of the real major axis radius. The larger radius of R1 and R2 is the best approximation, however. For non-orthogonal radius vectors including an angle of less than 45°, a circular cross-section was assumed by Wahle et al. [15]. In this case, the procedure is the same as for Method 1 and was automatically performed using an MS Excel macro. Finally, a Gambit journal file was generated and exported in IGES file format.

The ultimate generation of endo-luminal surface and volume geometry from cross-sections defined in IGES file format was done with the CAD-program SolidWorks™ (Solidworks Inc., Concord, USA). The IGES files were imported into SolidWorks™. The volume of each segment was then generated by interpolation using the "Loft" tool in SolidWorks™ with the segment centerline as a guide line. Bifurcations were modeled by extrusion. The branch segment was extruded toward the parent segment starting from the first cross-section of the branch segment until the start of the branch segment was completely inside of the parent segment. Figure 3 shows an example of a complete coronary artery reconstructed according to Method 1.

**Figure 4:**
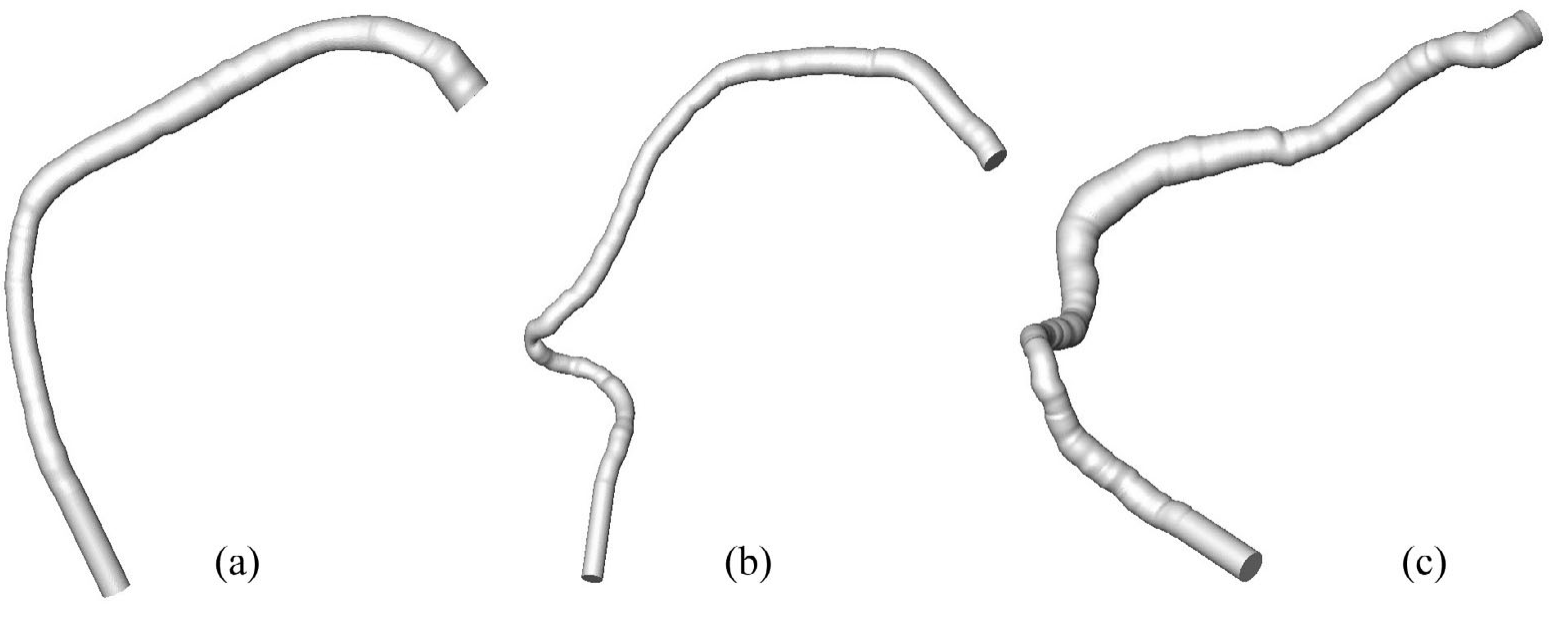
Endo-luminal surfaces of the three right coronary arteries reconstructed without branches: (a) – normal right coronary artery, (b) –, right coronary artery with "obstructive" atherosclerotic disease, and (c) – right coronary artery with "dilated" atherosclerotic disease. These geometries correspond to images shown in figure 1.

The CFD study described in this paper was limited to the reconstruction of the right coronary artery without branches (see figure 4). In order to smooth the outlet geometry for CFD and to eliminate the influence of outlet boundary conditions, we generated an additional 10 mm long segment by extruding the last cross-section of the reconstructed vessel in the direction normal to itself.

For the comparative study presented in this paper the following models were reconstructed: three HF models of three different coronary vessels with circular cross-sections, three HF models of three different coronary vessels with elliptical cross-sections, three LF models of three different coronary vessels with circular cross-sections and one DLF model of the normal coronary artery with a circular cross-section. Table 1 gives an overview of the main geometric parameters of the reconstructed segments.

**Table 1:** Geometric parameters of the three reconstructed right coronary arteries. D stands for diameter.

Parameters	Volume, mm^3^	Length, mm	Inlet D: mm	Outlet D: mm	Range of D: mm	Mean D: mm
"normal"	483	62	3.78	2.58	2.22 – 3.78	3.14
"stenosed"	413	94	2.97	2.19	1.78 – 2.97	2.36
"dilating"	1586	120	4.56	3.87	2.88 – 5.86	4.10

### Computational fluid dynamics

**Figure 5:**
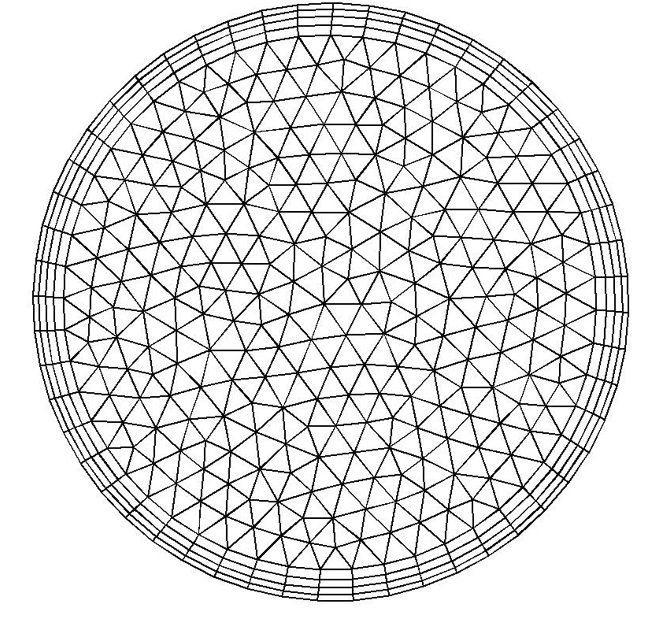
Grid in the inlet region of the right coronary model of the normal coronary artery.

The numerical solution of steady Navier-Stokes equations for momentum and mass conservation governing fluid motion under defined boundary conditions were solved by a control volume finite element method (FEM) implemented in FLUENT 6 (Fluent Inc., Lebanon, USA). For finite element numerical simulation the vessel volume had to be represented by a mesh grid. This transformation of the volume data was done by Gambit (software). The surface of the vessel was triangulated with a node distance between 0.1 and 0.2 mm (1:20 of the mean diameter). Based on this surface mesh, a grid composed of tetrahedral elements was generated in the reconstructed vessels. The total number of nodes exceeded 50,000. The average number of elements per cross-section was 350. Recently, some detailed studies were performed regarding the mesh resolution required to appropriately simulate the blood flow in coronary arteries using finite element methods [18,19]. The authors found that a high mesh resolution near the walls was needed in order to get accurate values of WSS. Based on the results of these studies [19,20], we generated a mesh which was refined in the near wall region. A boundary layer consisting of 4 rows, with a growth factor of 1.2 (ratio between two consecutive layers near the wall) and a total depth of 0.2 mm, was generated (see figure 5). The quality of the generated mesh grid was assessed using different approaches. The maximal skew of their distribution was, for example, below 0.75, which is fully satisfactory. The resulting number of grid volume elements ranged between 270,000 and 390,000 for the different vessels. Stationary laminar flow was simulated presuming rigid motionless walls. A no-slip condition was assumed at the wall. The pressure value was not imposed at the outlet. Blood was modeled as a Newtonian fluid with a kinematic viscosity of 3.5 10^-6 ^m^2^/s. A second order discretization scheme and a SIMPLE model for pressure flow coupling were used. A plug velocity profile was assumed at the inlet, because coronary arteries originate from a large compartment (sinus of the aortic root). The mean flow rate for each investigated vessel was estimated based on our flow rate measurements. These were recorded by the simultaneous measurement of pressure and velocity in these patients by a miniaturized ultrasound Doppler probe positioned within the coronary artery. The mean inlet velocities, 0.17 m/s for the normal patient, 0.27 m/s for the patient with "obstructive" coronary atherosclerosis and 0.09 m/s for the patient with "dilated" coronary atherosclerosis, were used, which resulted in the Reynolds numbers of 184, 228, and 117 respectively, for the three investigated geometries. The mass flows were 112.9 ml/min for the patient with a normal coronary artery, 111.8 ml/min for the patient with "obstructive" coronary artery disease and 88.5 ml/min for the patient with "dilated" coronary artery disease. The mass flows were calculated by multiplying the mean inlet velocity with the inlet cross-section areas obtained as the result of reconstructions. The smaller mass flow in the last patient is due to the smaller perfusion territory of the right coronary artery in this patient. The convergence criteria for relative errors in velocity components and pressure were set as 10^-5^.

### Statistics

In order to quantify the differences between the resulting WSS distributions, we generated distribution histograms. The whole range of calculated wall shear stress values was divided into 100 classes. For each class, the area corresponding to the WSS range was calculated and normalized as a percent of the total wall surface area. The sum of the calculated values from all classes was then equal to 100%. Cross-correlations and Chi-square tests were used to compare distribution histograms.

## Results

Three different coronary vessels were reconstructed using two different reconstruction methods (I and II) with a higher resolution – HF models. The reconstruction method I (circular cross-sections) was also used to generate LF models for all three coronary arteries. A DLF model was generated for the normal coronary vessel. Altogether, 10 models of three different coronary vessels were studied. The analysis of the reconstructed models shows that the number of cross-sections used in generating the high fidelity (HF) models resulted in a rather fine resolution of about 0.3–0.4 mm. This equals the resolution of the original data obtained by a reconstruction of biplane angiograms with software developed at the DHZB (German Heart Institute of Berlin). The resolution of low fidelity (LF) models was about 0.8 mm. However, the comparison of volumes and surfaces between HF and LF models yields an error of <1%.

**Figure 6:**
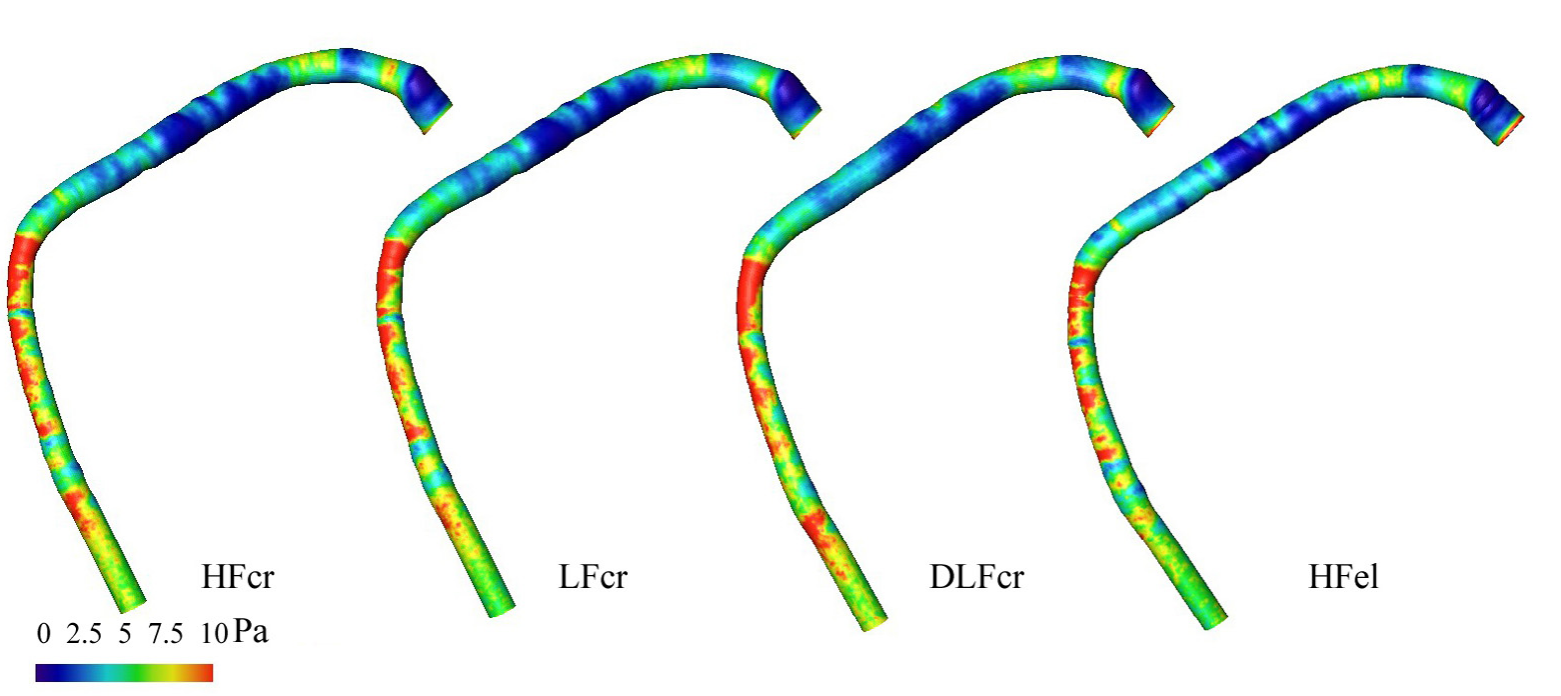
WSS distributions in the reconstructed normal coronary artery. From the left to the right: HF model reconstructed by Method 1, LF model reconstructed by Method 1, DLF model reconstructed by Method 1   and HF model reconstructed by Method 2 of the normal right coronary artery. The Reynolds number was Re = 184.

**Figure 7:**
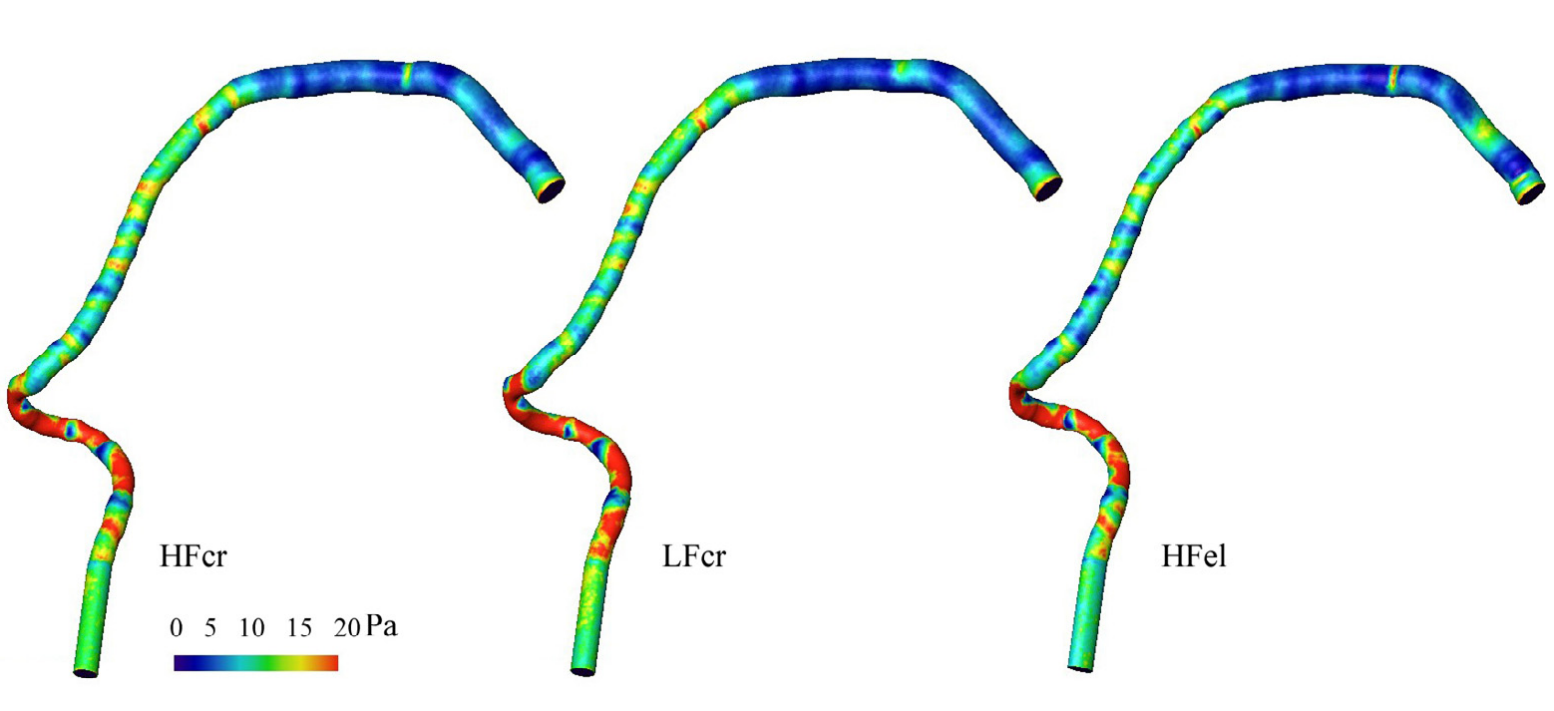
WSS distributions in the reconstructed right coronary artery with "obstructive" atherosclerotic disease. From the left to the right: HF model reconstructed by Method 1 (left), LF model reconstructed by Method 1 (middle), and HF model reconstructed by Method 2 (right) of the right coronary artery with "obstructive" atherosclerotic disease. The Reynolds number was Re = 228.

**Figure 8:**
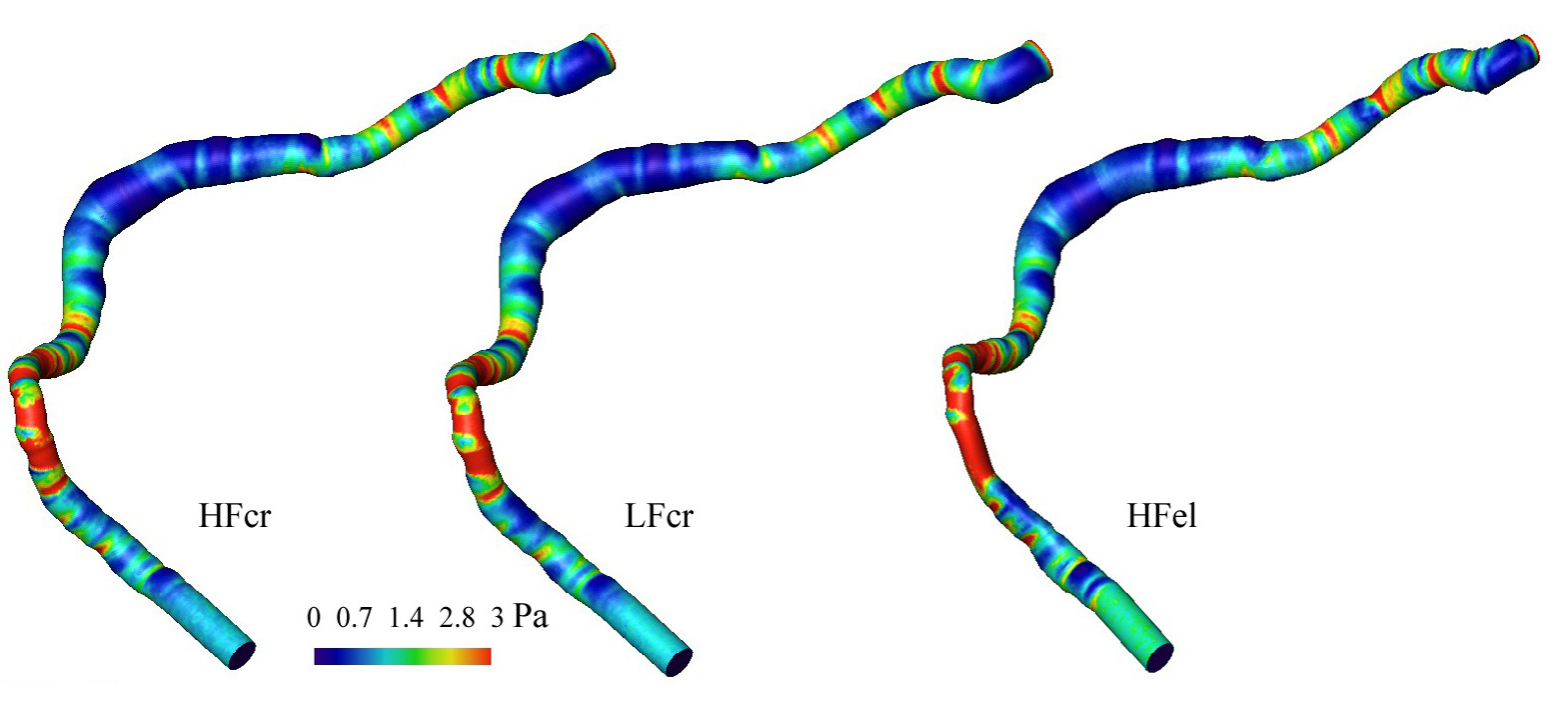
WSS distributions in the reconstructed right coronary artery with "dilated" atherosclerotic disease. From left to right: HF model reconstructed by Method 1 (left), LF model reconstructed by Method 1 (middle), and HF model reconstructed by Method 2 (right) of the right coronary artery with "dilated" atherosclerotic disease. The Reynolds number was Re = 117.

Steady numerical flow simulations of steady flow were done for the 10 reconstructed models of the three different coronary vessels. The corresponding distributions of WSS are visualized in figures 6, 7, and 8. The distributions of WSS for the reconstructions with high and low resolution appear identical.

**Figure 9:**
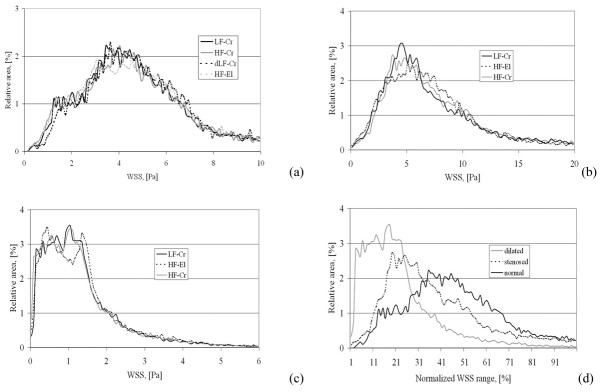
Images (a), (b) and (c) show histograms of the WSS distributions depicted in figures 6, 7 and 8 respectively. Image (d) shows a comparison of three histograms of the three different vessels with normalized WSS ranges for LF models reconstructed with Method 1. Cr stands for models with circular cross-sections. El stands for models with elliptic cross-sections.

Histogram curves were generated from calculated WSS distributions. The distribution curves agree well (see figures 9a, b and 9c), and demonstrate a strong cross-correlation (0.91 – 0.95) for each of the investigated three coronary arteries. In the Chi-square test for the comparison of histograms, no significant difference was found (p = 0.995).

The calculated pressure loss was slightly lower (2.5–8.5%) in LF models. Pressure drops strongly depended on the type of geometry (normal control: 6 mmHg, "obstructive" disease: 25.4 mmHg, or "dilated" disease: 1.83 mmHg).

The effect on the distribution of WSS, if a circular cross-section (Method 1) is assumed as opposed to an elliptic cross-section (Method 2), is also displayed in figures 6, 7, and 8. The comparison was performed for HF models. The distribution histograms shown in figures 9a, b and 9c are similar, and also demonstrate a high cross-correlation (0.87 – 0.94). Again, in the Chi-square test for the comparison of histograms, no significant difference was found (p = 0.995). The difference between the volumes was less than 0.017 ml (< 2%), the difference in wall area was less than 13 mm^2 ^(< 1%) and the pressure loss was slightly higher (3.6–5.3%) with the elliptical cross-sections in coronary artery disease. It should be noted that there are also some differences between the inlet diameters of models reconstructed with different methods (< 1.5 %). The differences between the outlet diameters of models reconstructed with different methods were even higher but remained below 5 %.

The three distinct varieties of coronary vessel geometry are characteristically reflected by the WSS histograms. The mean WSS was 4.6 Pa in the normal patient and the range was between 0 Pa and 10 Pa. The mean WSS was higher in the sample with "obstructive" atherosclerotic disease with 8.8 Pa, and had a wide range (0 Pa to 20 Pa). On the contrary, the mean WSS in the sample with "dilated" atherosclerotic disease was lower with 1.3 Pa, and had a rather narrow range (0 Pa to 6 Pa). However, the obtained WSS histogram curves also revealed characteristic shape differences (see figure 9d) which were shown by normalizing the WSS ranges. The histogram of the control patient is a symmetrically distributed curve with a single peak of WSS at 4 Pa that is nearly in the middle of the WSS range and is close to the mean value of 4.6 Pa (see figure 9a). These small differences between mean, median and peak values are also reflected in the low skew value for the histograms of the normal patient – 0.16. The histogram of the patient with "obstructive" atherosclerotic disease demonstrates an asymmetric distribution where the peak is at 5 Pa, which is located near to the peak seen in the histogram of the patient without coronary disease. However, there is a strong right-sided hump in the histogram curve (see figure 9b) which is assumed to correspond to stenotic parts of the vessel. This results in the rather large difference between peak (5 Pa), mean (8.8 Pa), and median (10 Pa) values of WSS. The asymmetry of the histogram curve of the patient with "obstructive" atherosclerotic disease is also reflected by a higher skew value – 0.7. The wide range of WSS values reflects multifocal disease and inhomogeneous remodeling. For the patient with "dilated" atherosclerotic disease, the histogram of WSS distribution is very asymmetric (one-sided), with the peak of WSS having shifted to low values at 0.9 Pa. This results in the strong difference between peak (0.9 Pa), mean (1.3 Pa) and median (3 Pa) values of WSS. The excessive asymmetry of the histogram curve of the patient with "dilated" atherosclerotic disease is reflected by a very high skew value – 0.97. The confinement of WSS to a narrow range of low values means diffuse negative remodeling. It should be noted that the simplified reconstruction approaches had no impact on the shape differences of these curves.

**Figure 10:**
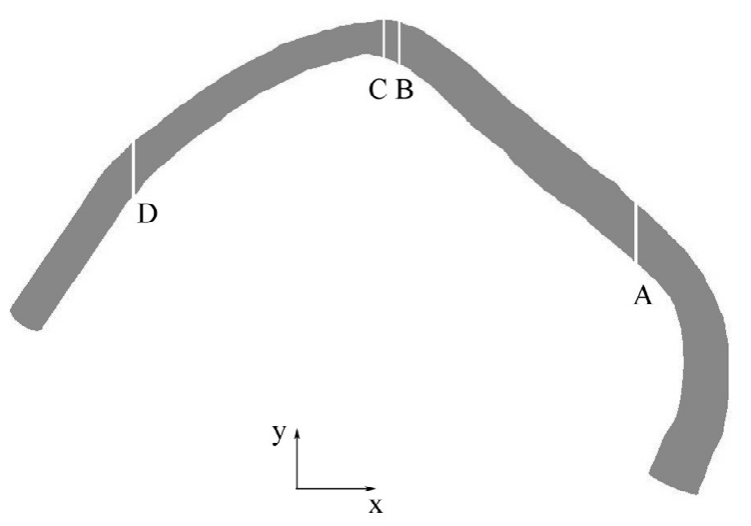
Normal coronary artery of the normal patient used for the analysis of the impact of evaluated simplifications on the local WSS distribution. The letter A marks the x position (x = 18 mm) of the first x-constant plane whereas the letter D marks the x position (x = -18 mm) of the last x-constant plane defining the region of the vessel wall used for the analysis of the local WSS distribution. Letters B and C mark two neighboring x-constant planes which define one of the 36 evaluated vessel wall parts.

**Figure 11:**
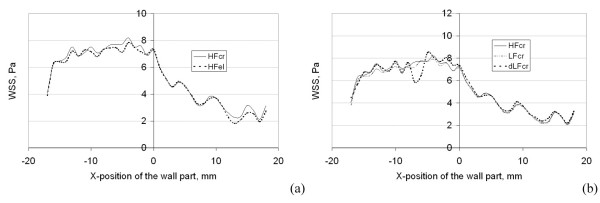
Comparison of the distribution of the WSS averaged over 36 local vessel wall sites for four of the models (the HF model reconstructed with Method 1 – HFcr; the LF model reconstructed with Method 1 – LFcr; the DLF model reconstructed with Method 1 – DLFcr and the HF model reconstructed with Method 2 – HFel) of the coronary artery of the control (normal) patient.

In order to quantify the effect of geometric simplifications on the local WSS distributions, we investigated the WSS distributions in the coronary artery of the normal patient, reconstructed by different methods, in more detail. A part of the wall surface of the normal patient coronary artery was divided by x-constant planes into 36 sections (see figure 10) for each of the three models (HF and LF models reconstructed with Method 1 and HF model reconstructed with Method 2). The distance between the planes was 1 mm. The mean WSS was calculated for each section. Figure 11 shows a comparison of the curves of the mean WSS values for different models along the x-axis (x-position). The difference in WSS values between HF and LF models (see figure 11b) reconstructed by Method 1, for all evaluated 36 sections, was 2.2% ± 1.7%. The maximal difference for one site was 6.6 %. The difference in WSS values between HF models reconstructed by Method 1 and Method 2 (see figure 11a) was 4.6% ± 5.3%. The maximal difference for one site was 14.3 %.

In order to assess the effect of further reduction of the spatial resolution of geometric reconstruction, we investigated the WSS distributions in the double low fidelity model (DLF) of the coronary artery of the normal patient reconstructed with Method 1. The mean distance between two cross-sections in this model was 1.6 mm. The comparison of volumes and surfaces between HF and DLF models yielded an error of about 1%. The corresponding distribution of WSS is visualized in figure 6. The distribution of WSS in the reconstructions with high and double low resolution appear identical in the proximal and middle parts of the coronary model. The differences in the distal part are more impressive. However, the distribution curves agree well (see figure 9a), and demonstrate a strong cross-correlation (0.93). In the Chi-square test for comparison of histograms, no significant difference was found (p = 0.995). The more detailed comparison of the local WSS values of the 36 vessel wall parts (middle part of the coronary artery) from the HF and DLF models revealed higher differences than between the HF and LF models reconstructed with Method 1 or than between the models reconstructed with Method 1 and Method 2. The difference in WSS values between HF and DLF models (see figure 11b) reconstructed by Method 1 for all 36 evaluated sections was 5.3% ± 5.2%. The maximal difference at any site was 26 %.

## Discussion

Wall shear stress is the most important mechanical regulatory signal which links flow to adaptive changes of the vascular wall and atherosclerotic lesions [21]. Three-dimensional reconstruction of coronary artery segments, with subsequent numerical flow simulation studies based on individual patient data, are currently the standard approach to in vivo flow profiling and WSS measurements in coronary arteries [1-9]. The prognostic clinical value of this approach is supported by the results of two recent clinical serial studies [8,9]. We do not know whether the costs of the enhanced accuracy of the complex modeling approach to WSS estimation proposed in [8] translates into an additional clinical diagnostic impact as compared to the simplified approaches [9]. A calculation based on a simplified geometric reconstruction would be an important step beyond exemplary studies from bench to bedside. Moreover, routine catheterization data, which do not supply the high resolution circumferential data (e.g. by IVUS) necessary for the complex approach, should be sufficient for a simplified model with a lower resolution. We investigated the impact of two simplifications of the geometric reconstruction of 3-D vessel lumina on the accuracy of flow simulation in coronary arteries.

Using a LF model reduces the spatial resolution by a factor of two; however, it also reduces the time for the 3-D geometry reconstruction by nearly a factor of 3. This time saving results from the fact that reconstruction problems due to intersecting cross-sections are avoided. The low resolution (LF) model demonstrated a negligible impact on the vessel wall area and the WSS distribution as compared to the results for the HF model. The differences in pressure drops between the HF and LF model were small compared to the differences related to the type of geometry (normal, obstructive or dilated). The deviation of the distribution histograms, caused by the reduced resolution in the LF model, was not significant and small compared to the differences due to the type of geometry. Hence, the HF resolution neither improves the results nor adds additional clinical information. Consequently, LF models are preferred. The LF models smooth the surface geometry. As a result, some local information is lost that may be important. Since the difference between HF and LF models is equivalent to a reduction of the spatial resolution by a factor of two, we studied the impact on the accuracy of the local geometric variability (see figure 11) and found that a sufficient accuracy of reconstruction is preserved. The LF model has a mean distance of 0.8 mm between the two cross-sections used for reconstruction. This resolution of the vessel reconstruction along the vessel axis is of the same order as the slice thickness of the standard CT devices and better than the resolution of IVUS and MRI imaging. Further reduction of the spatial resolution by a factor of four in the DLF model revealed a significant loss of local information about the WSS distribution.

The comparison of HF models of coronary arteries, reconstructed by Method 1 and Method 2, demonstrated only minor differences in the vessel wall area and the WSS distribution. The maximal error of the calculated pressure drop was 6.8 % for simulations in the coronary artery of the normal patient, which is much lower than the variability of the pressure drop in the different types of geometry. The deviations in the histograms of WSS were much larger between the three coronary arteries with a different geometry than the variability caused by the reconstruction method. On the other hand, the differences caused by the reconstruction method were higher than the differences caused by the reduction of model resolution. This is reflected by the lower cross-correlation coefficients (0.91–0.95) that were found by analyzing the effect of resolution and 0.87–0.94 that were found by analyzing the effect of the reconstruction method. One reason for the deviation of the pressure drop between different models of the same vessel is the use of hydraulic radii (radii calculated from the cross-sectional area) in the elliptical cross-sections with non-orthogonal radii. The geometric mean radius is equal to the hydraulic radius only if the radii are orthogonal. For example, the inlet diameters of the coronary models reconstructed with Method 1 were 3.78 mm in the coronary artery of the control patient, 2.96 mm in the coronary artery of the patient with "obstructive" disease and 4.54 mm in the coronary artery of the patient with "dilated" disease. The hydraulic diameters of the coronary models reconstructed with Method 2 were 3.73 mm, 2.97 mm, and 4.53 mm respectively. Note that a difference of 1% in the vessel radius causes a difference of about 2% in the pressure drop for the same inlet velocity. The second, and more important, reason for the differences in pressure drop and WSS caused by reconstruction Method 2, as opposed to Method 1, are irregularities in the cross-sections reconstructed with Method 2. For non-orthogonal radius vectors including an angle of less than 45°, a circular cross-section was assumed by Wahle et al. without any interpolation or smoothing with respect to cross-sections in the neighborhood [15]. A circular cross-section, instead of an elliptic one, occurs in about 30% of all the cross-sections (3000 cross-sections were analyzed). These artificial wall irregularities cause artifactual local flow disturbances and WSS artifacts that are avoided by the smoother reconstruction technique (Method 1), which uses a circular shape for all reconstructed cross-sections. Thus, the use of Method 1 for geometry reconstruction should provide fewer artifacts, and hence, more accurate and realistic results from the CFD calculations. Furthermore, Method 1 is more suitable for further automatization of the reconstruction algorithm and may be implemented by macro programming in SolidWorks™. Last but not least, Method 1 reflects the physiology of luminal remodeling which is highly localized and preserves a circular lumen even in the majority of eccentric atherosclerotic lesions [12].

The problems of the geometry reconstruction using the software developed by DHZB (intersecting cross-sections and irregularities in cross-sections and therefore irregularities in the interpolated surface caused by the use of elliptical and circular shapes) are mainly due to the fact that the primary goal of the software was an assessment of the vessel diameters and volumes, and that a further use for numerical flow simulations was not considered at that stage. The above mentioned problems may be alternatively resolved by an algorithmic approach. However, this implies a time-consuming modification of the existing software. The proposed simplifications for studying WSS profiling in a coronary reconstruction, allows immediate and statistically relevant profiling in a larger sample of available data (about 60 coronary arteries).

Since the geometry is the main factor influencing the WSS distribution, it is important to consider the impact of neglecting branch flows. Surprisingly, it was shown that branch flows are of minor importance in determining WSS patterns in the trunk of the right coronary artery [18].

One of the important issues of WSS profiling is the validation of WSS profiling based on biplane angiograms. Unfortunately, we can not consider 3-D-IVUS as a gold standard for our method. None of these methods provides a direct measurement. IVUS provides a better resolution for an assessment of the vessel cross-section. However, the resolution is worse with regard to the reconstruction of geometry along the vessel axis in the main flow direction. Furthermore, IVUS is not suitable for studying complex vessel trees similar to the vessels shown in figure 2.

Further simplifications in our study concerned the numerical model. The momentum and mass conservation equations have to be solved under difficult boundary conditions in order to fully model the blood flow in arteries. All physiological parameters have to be accounted for, i.e. wall compliance, pulsatile flow, and non-Newtonian behavior of the blood. In addition, in a coronary artery, cardiac contraction induces a continuous, site specific motion and deformation of the vessel. All these aspects may affect flow patterns and were thoroughly studied in the last years [18,22-26]. The impact of the assumption of rigid or non-rigid arterial walls has been well investigated and discussed in the literature [22,23]. The authors of these studies agree that the assumption of a rigid wall is sufficiently accurate for WSS profiling in investigating atherosclerosis for clinical purposes. This judgment is based on their calculations with regard to reconstructions of the first bifurcation of the left coronary artery. However, these results are also valid for right coronary arteries, since there are no significant differences between these two arteries from a hemodynamic point of view (Reynolds and Strouhal numbers, Womersley parameter, pressure pulse wave and vessel thickness). Among the deformations of the coronary arteries due to cardiac surface motion, only torsion is assumed to have a small effect on local WSS. The effect of pulsatility was also found to be small and to have a limited effect on local WSS. No differences were found between steady-state calculations of the WSS distribution and time-averaged calculations over a whole heart cycle [18,26]. Average calculated Womersly parameters (Wo = R(2πf/ν)^0.5^, where R is the artery radius, f is the heart frequency and ν is the kinematic viscosity of blood) were found to be low in coronary arteries (Wo = 3.05 ± 1.00, N = 117) due to the small radii of the coronary arteries (own unpublished results based on in-vivo coronary hemodynamic data and quantitative coronary angiography data acquired by cardiac catheterization). Since flow unsteadiness associated with pulsatility has a significant impact on the local WSS only, if Wo>>1 (cut-off 5). Pulsatile flow modeling is not necessary in coronary arteries, if we study time-averaged WSS. The WSS distribution averaged over one heart cycle is considered as the main hemodynamic parameter that links flow to adaptive changes of the vascular wall and atherosclerotic lesions. There are other parameters, which are held to be important: e.g. temporal (WSSGt) and spatial WSS gradients (WSSG), and the oscillating WSS index (OSI) [18]. Many of the above-mentioned aspects (e.g. pulsatility, wall elasticity, non-Newtonian blood behavior), do apply for these parameters (OSI, WSSGt, WSSG). Last but not least, it should be noted that atherosclerotic wall alterations reduce wall elasticity and eventually lead to a rigid wall model.

The most interesting findings of this study are the differences between the WSS histogram curve shapes. These differences seem to be characteristic for these three different coronary arteries which represent three different entities of coronary pathology (normal patients, patients with "obstructive atherosclerosis" and patients with "dilated" atherosclerosis) and might have diagnostic value. However, further studies with a larger number of coronary arteries are necessary to assess the clinical value of these findings. Local information about WSS distribution may be smoothed by our approach. Such local information is thought to be very important for the study of the correlation between WSS distribution and the distribution of atherosclerotic wall alterations or intimal thickening. However, a low WSS value does not imply that wall alterations will necessarily be present at the corresponding site. The relation between WSS and biologic vascular response is modulated by many other factors. The assessment of this relation needs a probabilistic and multi-factorial approach. We know that clinically symptomatic coronary atherosclerosis usually shows a multimodal, or even diffuse distribution, and demonstrates distinct varieties of positive or negative remodeling associated with different geometries. According to the statistical principle of stratified sampling, extreme varieties of coronary geometries were selected to identify characteristic features of the impact of global geometry on flow and WSS. A characteristic global parameter is mean WSS. The mean WSS may be estimated from the mean vessel radius R and the mean velocity V as τ = μ(4 V/R) (Eq. 1), where μ is the dynamic blood viscosity. Using this equation, the mean WSS may be calculated as 1.49 Pa for the coronary of the normal patient, 2.95 Pa for the coronary from the patient with "obstructive" atherosclerotic disease and 0.6 Pa for the coronary from the patient with "dilated" atherosclerotic disease. The average WSS values calculated from the CFD results are 4.6 Pa, 8.8 Pa and 1.3 Pa respectively. Thus a simplified approach based on the mean radius and mean velocity results in significantly lower values of WSS as compared to values based on the CFD calculations. Moreover, the information of the WSS histograms on scatter, distribution, skew, and peak values of WSS provides quantitative information on the diffuseness, the inhomogeneity and the progress of the atherosclerotic disease and the extent and type of associated remodeling as reflected by the resultant luminal geometry. It should be emphasized that the histograms obtained from the CFD solution characterize a very complex flow pattern, including flow separations and flow recirculations caused by the vessel curvature and local narrowings or enlargements. Such histograms cannot be obtained by a simplified approach as the calculation of WSS for each reconstructed cross-sectional volume slice is based on the Hagen-Poiseuille equation (Eq. 1). Furthermore, the WSS estimation, by using the Hagen-Poiseuille equation, is not able to predict the existence of the wall areas with low wall shear stress values (τ < 0.5 Pa), as it is done by the CFD results. These values are included in our histograms. These low WSS values are very important for our study, since they correlate with the loss of endothelial function and WSS responsiveness [13,28].

The distinct differences in the distribution of WSS values obtained from histograms may help to distinguish between, and assess the severity of, different coronary artery diseases. Further analysis is currently being performed in our 3-D coronary database consisting of right and left coronary arteries from 6 control patients, 10 patients with "obstructive" atherosclerotic disease, and 8 patients with "dilated" atherosclerotic disease.

There were a lot of investigations which considered the problem of reconstruction procedure on the accuracy of the numerical flow simulation in vessels, and especially for coronary arteries [18,20,22]. The novel aspects of the presented study are the following: we used a non-dimensional allometry approach (WSS histogram curve) to characterize and compare WSS in different coronary arteries, we studied the impact of two reconstruction simplifications on wall shear stress characterization using the WSS histogram curve, we used circular instead of elliptical cross-sections, and we used a reduced number of cross-sections for volume reconstruction. The study was applied to three different right coronary arteries representing three different geometries of this vessel: a right coronary artery of a control patient, a right coronary of a patient with "obstructive" atherosclerotic disease and a right coronary of a patient with "dilated" atherosclerotic disease. The study of three different vessels allowed us to show that the proposed simplifications are not significant for the differentiation of these coronary arteries by the proposed method.

As we mentioned above, there are no significant differences between right and left coronary arteries from a hemodynamic point of view. Hence, the results obtained in this study with right coronary arteries should also be valid for studies with left coronary arteries.

## Conclusion

A simplified approach to the reconstruction of coronary vessel lumina from biplane angiograms, by assuming circular cross-sections and using only every second vector triplet of the original data with the mean distance of 0.8 mm between cross-sections, yields sufficiently accurate calculations of vessel volumes, surfaces, wall shear stress distributions, and pressure drops. The resolution of 0.8 mm is accurate enough for the global (histogram) and local characterization of the wall shear stress in coronary arteries. This is valid also for other geometry reconstruction methods (3-D-IVUS or magnet resonance imaging). Lower resolution results in non significant deviations for the global characterization parameter of the WSS and in significant local alterations in WSS calculations. The issue, which remains to be validated, is: May we use biplane angiograms for WSS profiling? A final decision on this subject requires a study with a phantom of a real coronary artery, for which an exact computer model exists. This study is under way in our group.

Profiles of WSS within whole segments or vessels might identify different patterns of remodeling associated with characteristic changes in the distribution of WSS and quantify the extent and diffuseness of coronary artery disease. However, this issue needs further investigation in a larger number of patients with different coronary geometries in each group (controls, "obstructive" and "dilated" coronary atherosclerosis).

## Competing interests

The author(s) declare that they have no competing interests.

## Authors' contributions

Dr. Wellnhofer from German Heart Institute of Berlin performed biplane patient angiograms and velocity measurements with a miniaturized ultrasound Doppler probe. He is also one of the developers of the reconstruction software. Dr. Goubergrits developed the reconstruction software further that allowed performing a volume reconstruction, which may be used for numerical simulations with a CFD code. He performed also the numerical simulations with CFD code FLUENT and proposed the analysis of the WSS distributions by the WSS histograms. Dr. Kertzscher was involved in the statistical analysis of the obtained results. Prof. Dr. Affeld is a supervisor of the project and was also involved in the analysis and the discussion of the obtained results.
